# Elevated Serum Level of Cytokeratin 18 M65ED Is an Independent Indicator of Cardiometabolic Disorders

**DOI:** 10.1155/2020/5198359

**Published:** 2020-03-31

**Authors:** Lingling Qian, Lei Zhang, Liang Wu, Jing Zhang, Qichen Fang, Xuhong Hou, Qiongmei Gao, Huating Li, Weiping Jia

**Affiliations:** ^1^Department of Endocrinology and Metabolism, Shanghai Jiao Tong University Affiliated Sixth People's Hospital, Shanghai Key Laboratory of Diabetes Mellitus, Shanghai Clinical Center of Diabetes, Shanghai 200233, China; ^2^Department of Medicine, Shanghai Jiao Tong University School of Medicine, Shanghai 200025, China

## Abstract

**Background:**

Recent studies have suggested that cell death might be involved in the pathophysiology of metabolic disorders. The cytokeratin 18 (CK18) fragment, as a cell death marker, plays an important role in nonalcoholic fatty liver disease (NAFLD). However, only a limited number of studies have found elevated serum levels of CK18 in patients with type 2 diabetes. Moreover, no studies have been conducted yet to investigate the role of CK18 in hypertension or dyslipidemia. In particular, CK18 M65ED is a more sensitive marker of cell death, and its role in cardiometabolic disorders has not been revealed yet.

**Methods:**

A total of 588 subjects were enrolled from the local communities of Shanghai. Serum CK18 M65ED were determined using the enzyme-linked immunosorbent assay. A cardiometabolic disorder was identified by the presence of at least one of the components including overweight or central obesity, diabetes, dyslipidemia, and hypertension.

**Results:**

Subjects with cardiometabolic disorders exhibited significantly higher serum levels of CK18 M65ED than those without cardiometabolic disorders (197.36 (121.13–354.50) U/L versus 83.85 (52.80–153.75) U/L, respectively, *P* < 0.001). Increased serum CK18 M65ED quartiles were associated with the increased prevalence of cardiometabolic disorders and its components (*P* < 0.001 for all components). Multiple stepwise regression analysis also revealed that diastolic blood pressure, glycated hemoglobin A1c, alanine transaminase, and high-density lipoprotein cholesterol were independently correlated with serum CK18 M65ED levels (all *P* < 0.01). In addition, logistic regression analysis showed that the serum CK18 M65ED levels were positively correlated with cardiometabolic disorders and in an independent manner. Further, CK18 M65ED was revealed to be an indicator of cardiometabolic disorders in a NAFLD-independent manner.

**Conclusions:**

Elevated levels of CK18 M65ED, a sensitive cell death marker, were independently and positively correlated with cardiometabolic disorders, even after the adjustment for the presence of NAFLD and other cardiovascular risk factors.

## 1. Introduction

Cardiovascular disease (CVD) remains the leading cause of death and disability in developed countries, and it accounted for the death of approximately 17 million people worldwide in 2013 [1]. Cardiometabolic disorders, including obesity, type 2 diabetes (T2D), insulin resistance (IR), hypertension, and dyslipidemia, are common risk factors for CVD [2]. Moreover, the steady increase in the occurrence of cardiometabolic disorders compels the search for the involved pathophysiology and more effective preventive strategies.

Cytokeratin 18 (CK18) is involved in cell death pathway, and it is an important cell death marker. Cell death (apoptosis, necrosis, autophagy, etc.) can help to get rid of damaged cells and protect cell integrity when a cell responds to the aberrant cellular stresses like endoplasmic reticulum (ER) stress and oxidative stress, which have been the hallmarks of cardiometabolic disorders [3–5]. Massive beta cell death [6, 7] and cell death-induced decrease in the delta cell volume and number cause T2D [8]. Endothelial apoptosis can lead to endothelial dysfunction and development of hypertension [9]. Apoptosis in response to glucose excess was observed in cardiometabolic diseases [10], and decreased myocardial levels of apoptosis were found to significantly reduce obesity-associated cardiomyopathy [11]. The cellular content of the soluble fraction of CK18 has been shown to be released into the extracellular space during cell death both in vitro and in vivo [12]. A recent study indicated that CK18 played a role in apoptosis, which may be mediated via a feedback regulation loop and may involve regulation of transcription and alternative splicing of a number of genes in NF-*κ*B pathway and certain apoptosis pathways [13]. In addition to caspase-induced apoptosis, other forms of cell death can lead to the release of CK18. For example, CK18 is released during pyroptosis, nonlethal processes of apoptosis independent of caspase activation, or secondary lysis following programmed cell death [14, 15].

However, limited studies have focused on the role of serum CK18 in nonalcoholic fatty liver disease (NAFLD) and cardiometabolic disorders. Serum CK18 was found to be closely associated with hepatic inflammation in patients with NAFLD [16–18]. Chang et al. found that elevated levels of CK18 (assessed by M30 and M65 assays) in patients with T2D had a significant positive association with fasting plasma glucose levels, and it was independent of NAFLD status [19]. Tan et al. demonstrated the correlation between elevated levels of CK18 M30 and IR in patients with polycystic ovary syndrome [20]. Civera et al. showed that increased insulin resistance induces an increase in the levels of CK18 M30 in patients with severe obesity [21]. M30 was highly correlated to diastolic dysfunction indices in adolescents with obesity [22]. However, the role of CK18 in hypertension or dyslipidemia in adults has not been studied yet. Furthermore, the association between serum CK18 M65ED levels and cardiometabolic disorders has also not been investigated. In the present study, we therefore investigated this association with a focus on cardiometabolic disorders including overweight or central obesity, diabetes, dyslipidemia, and hypertension.

## 2. Methods

### 2.1. Study Participants

Subjects aged 18 years or older were enrolled from the local communities in Shanghai. All subjects underwent comprehensive physical examinations, routine biochemical analyses of blood, 75 g oral glucose tolerance test, hepatitis B surface antigen test, hepatitis C virus antibody test, and B-ultrasonography. The histories of present and past illnesses, medical therapy, and alcohol consumption were obtained through standard questionnaires. Subjects with the following conditions were excluded from this study: biliary obstructive disease, acute or chronic viral hepatitis, drug-induced liver disease, autoimmune hepatitis, Wilson's disease, known hyperthyroidism or hypothyroidism, presence of cancer, ongoing treatment with systemic corticosteroids, pregnancy, and current drinkers and exdrinkers. A total of 588 subjects were enrolled in the study. Of them, 310 had cardiometabolic disorders, and the control group consisted of 278 age-matched participants without conditions of obesity, central obesity, diabetes, dyslipidemia, or hypertension. The study was approved by the Ethics Committee of Shanghai Jiao Tong University Affiliated Sixth People's Hospital and was conducted in accordance the principles of the declaration of Helsinki. All data were handled without compromising the privacy of the study participants. All participants provided written informed consent prior to enrollment.

### 2.2. Anthropometric and Laboratory Measurements

**Figure 1:**
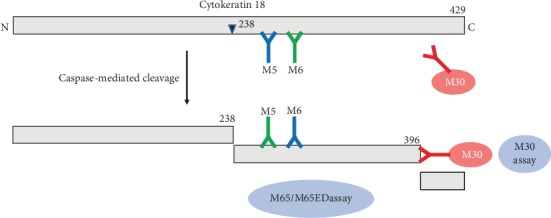
The difference between CK18, CK M30 assay, CK M65 assay, and CK M65ED assay.

Blood pressure, weight, height, waist circumference (WC), and biomedical indices were measured according to our previous standardized protocols [23]. Body mass index (BMI) = weight (kg)/height^2^ (m^2^). Blood samples collected from the subjects following an overnight fast of at least 10 h were tested to measure the levels of serum fasting plasma glucose (FPG), fasting insulin (FINS), glycated hemoglobin A1c (HbA1c), alanine transaminase (ALT), aspartate aminotransferase (AST), gamma-glutamyl-transferase (GGT), triglyceride (TG), total cholesterol (TC), high-density lipoprotein cholesterol (HDL-C), and low-density lipoprotein cholesterol (LDL-C). The 2-hour plasma glucose (2hPG) levels were measured following a 75 g oral glucose tolerance test (OGTT). Basal insulin secretion and insulin sensitivity were calculated using the homeostasis model assessment (HOMA) calculations: HOMA − B = [(FINS) (mU/L) × 6 − 3.33]/[FPG (mmol/L) − 3.5] and HOMA − IR = FINS × FPG/22.5 [23]. The concentration of serum CK18 M65ED was quantified using the M65 EpiDeath enzyme-linked immunosorbent assay kit (Peviva AB, Bromma, Sweden). As shown in Figure 1, the difference between CK M30 assay, CK M65 assay, and CK M65ED assay was explained. We chose the M65ED assay in this present study as a more sensitive marker for cell death. The intra- and interassay variations for the measurement of CK18 M65ED were 5.23% and 7.12%, respectively.

Cytokeratin 18 is a major intermediate filament protein of the cytoskeleton that is expressed in simple epithelial cells, hepatocytes, cholangiocytes, pancreas, and colon [16, 24, 25]. During apoptosis, full-length CK18 can be cleaved at two sites into three fragments [26]. The M30 assay can detect the caspase-cleaved fragments of CK18 by using the monoclonal antibody, M30, showing the extent of apoptosis [24]. The CK18 M65 assay uses the capture antibody M6 and the detection antibody M5 that are directed against two different epitopes of CK18 and can recognize both full-length CK18 and cleaved fragments of CK18, regardless of whether they are cleaved by caspases or not [18]. The M65ED assay uses the M6 antibody for detection and the M5 as a capture antibody, which can achieve improved binding specificity and lower signals in healthy controls [24, 27].

### 2.3. Diagnostic Criteria

Cardiometabolic disorders were identified by the presence of at least one of the components including overweight or central obesity, diabetes, dyslipidemia, and hypertension [28]. An overweight condition was defined as a BMI between 25.0 and 29.9 kg/m^2^, and obesity was defined as a BMI ≥ 30 kg/m^2^ [29]. Central obesity was defined as a waist circumference ≥ 90 cm for Chinese men and ≥80 cm for Chinese women [30]. For diabetes, a self-reported diagnosis of diabetes determined previously by a health care professional was considered in addition to FPG ≥ 7.0 mmol/L, 2hPG ≥ 11.1 mmol/L during OGTT, HbA1c ≥ 6.5%, subjects with classic symptoms of hyperglycemia or hyperglycemic crisis, and random plasma glucose ≥ 11.1 mmol/L [31]. Dyslipidemia condition was defined as the levels of TC ≥ 6.20 mmol/L, TG ≥ 2.26 mmol/L, HDL − C < 1.03 mmol/L, and LDL − C ≥ 4.13 mmol/L, or taking lipid-lowering medications [32]. Hypertension condition was defined as a systolic blood pressure (SBP) ≥ 140 mmHg, diastolic blood pressure (DBP) ≥ 90 mmHg, or taking blood pressure-lowering medications [33]. The guidelines for the diagnosis of NAFLD proposed by the Asia-Pacific Working Party were used [34]. NAFLD was clinically defined as manifestations of B-ultrasonography performed by experienced radiologists, ruling out the drinking habit, and the specific diseases that could result in fatty liver, according to our previous standardized protocols [23].

### 2.4. Statistical Analysis

All analyses were performed using SPSS 25.0 (Chicago, IL, USA). Normally distributed data were expressed as mean ± SD. Data that were not normally distributed were log-transformed before analysis and expressed as median with interquartile range. Categorical variables were expressed as a percentage (%). Student's unpaired *t*-test and chi-square test were used for comparison between two groups. Analyses were performed separately for different genders and different components of cardiometabolic disorders. Pearson's correlation and multiple stepwise regression analysis were used to examine the associations between serum CK18 M65ED levels and various parameters. Multiple logistic regression was used to assess the association of serum CK18 M65ED levels with the risk for cardiometabolic disorders. Serum CK18 M65ED levels were entered in the following two ways: as quartiles and as a continuous variable. The odds ratio (OR) was calculated to determine whether the relevant factors were risk factors for cardiometabolic disorders. The number of cardiometabolic disorders for each subject was indicative of the total number of each cardiometabolic disorder component: obesity or central obesity, diabetes, dyslipidemia, and hypertension. Hence, for each cardiometabolic disorder component, the subjects received a 1 if it was present or a 0 if not present. A two-tailed *P* value <0.05 was considered indicative of a statistically significant difference.

## 3. Results

### 3.1. Clinical Characteristics of the Study Subjects

A total of 588 subjects were enrolled in this study (median age: 40.00 (33.00–49.75) years) and included 340 men and 248 women.  Table 1 lists the clinical and laboratory characteristics of the subjects. Subjects with cardiometabolic disorders (*n* = 310) exhibited higher values for BMI, WC, SBP, DBP, 2hPG, HbA1c, FINS, HOMA-IR, HOMA-B, ALT, AST, GGT, TG, TC, and LDL-C (all *P* < 0.05) and a lower value for HDL-C (*P* < 0.001) than those without cardiometabolic disorders (*n* = 278). Age and FPG did not differ significantly between the two groups (all *P* > 0.05).

### 3.2. Serum CK18 M65ED Levels in Different Components of Cardiometabolic Disorders

**Figure 2:**
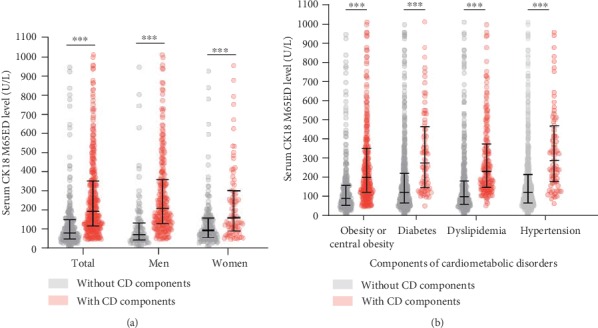
Comparison of serum CK18 M65ED levels between patients (a) with and without cardiometabolic disorders; (b) in the subgroup analysis regarding the presence of each of the different components of the cardiometabolic disorders. Data are shown as median with 25th and 75th percentiles. ^∗∗∗^*P* < 0.001, CD versus without CD. CD: cardiometabolic disorders; CK18: cytokeratin 18.

As shown in Table 1, the median serum CK18 M65ED level for all subjects was 137.54 (76.51–254.39) U/L. The subjects with cardiometabolic disorders showed higher serum CK18 M65ED levels (197.36 (121.13–354.5)) than those without cardiometabolic disorders (83.85 (52.8–153.75) U/L) (*P* < 0.001). There was a significant difference in the serum CK18 M65ED levels between men and women with and without cardiometabolic disorders (211.60 (132.53–361.30) U/L versus 74.70 (48.22–136.57) U/L in men, respectively, *P* < 0.001 and 161.96 (98.33–301.95) U/L versus 96.10 (60.83–158.93) U/L in women, respectively, *P* < 0.001; Figure 2(a)). Serum CK18 M65ED levels were higher in subjects with obesity or central obesity (202.86 (124.18–350.75) U/L) than nonobese subjects (93.66 (57.82–160.6) U/L). Serum CK18 M65ED levels were higher in subjects with diabetes (279.63 (151.59–457.59) U/L) than nondiabetic subjects (124.43 (69.47–223.53) U/L). Serum CK18 M65ED levels were higher in the subjects with dyslipidemia (233.98 (152.82–371.84) U/L) than those without dyslipidemia (103.41 (60.89–184.01) U/L). Serum CK18 M65ED levels were higher in the subjects suffering from hypertension (291.61 (182.44–460.18) U/L) than those not suffering from hypertension (124.58 (69.7–218.12) U/L) (all *P* < 0.001; Figure 2(b)).

### 3.3. Clinical Characteristics and Prevalence of Cardiometabolic Disorders Stratified by Quartiles of Serum CK18 M65ED Levels

**Figure 3:**
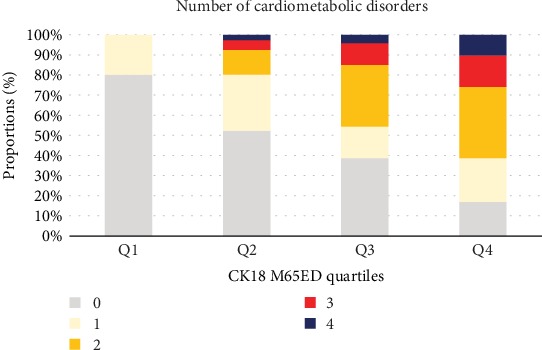
Proportions of participants with multiple cardiometabolic disorders according to serum CK18 M65ED quartiles. Histograms are weighted percentages. The number of cardiometabolic disorders for each subject was the total presence number of obesity or central obesity, diabetes, dyslipidemia, and hypertension; that is, for each cardiometabolic disorder component, the participants received a 1 if this disorder was present, and 0 otherwise. CK18: cytokeratin 18.

The CK18 M65ED quartiles were defined according to the median and interquartile values for the serum CK18 M65ED levels for the entire study population as follows: quartile 1, ≤76.51 U/L (*n* = 147); quartile 2, 76.51–137.54 U/L (*n* = 147); quartile 3, 137.54–254.39 U/L (*n* = 147); and quartile 4, ≥ 254.39 U/L (*n* = 147). Clinical parameters, such as BMI, WC, SBP, DBP, FPG, 2hPG, HbA1c, FINS, HOMA-IR, ALT, AST, GGT, TG, TC, and LDL-C, displayed an increasing trend from quartile 1 to quartile 4 (all *P* < 0.001; Table 2). However, HDL-C displayed a decreasing trend from quartile 1 to quartile 4 (*P* < 0.001; Table 2). In addition, the prevalence of cardiometabolic disorders and its components (obesity, central obesity, diabetes, dyslipidemia, and hypertension) increased from quartile 1 to quartile 4 (all *P* < 0.001; Table 2). As shown in Table 2 and Figure 3, the number of cardiometabolic disorder components also increased from quartile 1 to quartile 4.

### 3.4. Association of Clinical Parameters with Serum CK18 M65ED Levels

Pearson's correlation analysis revealed positive correlations between BMI, WC, SBP, DBP, FPG, 2hPG, HbA1c, HOMA-IR, ALT, AST, GGT, TG, TC, and LDL-C, and serum CK18 M65ED levels (all *P* < 0.001) (Table 3). HDL-C showed a negative correlation with serum CK18 M65ED levels. To further identify factors independently affecting serum CK18 M65ED levels, we performed stepwise regression analysis with the serum CK18 M65ED levels designated as the dependent variable and BMI, WC, SBP, DBP, FPG, 2hPG, HbA1c, HOMA-IR, ALT, AST, GGT, TG, TC, HDL-C, and LDL-C levels designated as the independent variables, with the adjustment for gender. The analysis revealed that the levels of DBP (standardized *β* = 0.207), HbA1c (standardized *β* = 0.171), ALT (standardized *β* = 0.474), and HDL-C (standardized *β* = −0.135) were independently associated with serum CK18 M65ED levels (all *P* < 0.01).

### 3.5. Serum CK18 M65ED Is an Independent Risk Factor for Cardiometabolic Disorders

We performed a binary logistic regression analysis in which the presence of cardiometabolic disorders was designated as the dependent variable in three different models, and adjustments were made for age, gender, BMI, and other related clinical parameters. The multivariable-adjusted (age, gender, and BMI, model 1) OR of cardiometabolic disorders across the quartiles of CK18 M65ED were 1.00 (95% confidence interval (CI), 1.00–1.00), 2.77 (95% CI, 1.31–5.85), 3.14 (95% CI, 1.43–6.89), and 9.08 (95% CI, 3.91–21.08) (all *P* < 0.001) (Table 4). The association was still significant (*P* = 0.012) after further adjustment for SBP, DBP, ALT, AST, GGT, TG, TC, HDL-C, LDL-C, HOMA-IR, HOMA-B, and HbA1c (model 2) and maintained significant difference (*P* = 0.018) after adjustment for SBP, DBP, ALT, AST, GGT, TG, TC, HDL-C, LDL-C, FPG, 2hPG, HbA1c, and FINS (model 3). Considering that CK18 levels are elevated in NAFLD, we adjusted the binary variable of NAFLD with age and gender, and still observed significant association (*P* = 0.011, model 4), suggesting that the association of CK18 with cardiometabolic disorders is independent of NAFLD. Upon examining CK18 M65ED levels as a continuous variable, each 1 SD increase in the CK18 M65ED levels was a positive predictor of cardiometabolic disorders (all *P* < 0.05) with the adjustment of different variables in different models.

## 4. Discussion

In the present study, we have demonstrated that serum CK18 M65ED levels are significantly increased in Chinese subjects with multiple cardiometabolic disorders and these levels positively correlate with DBP, HbA1c, ALT, and HDL-C levels. Our analyses showed that increased serum CK18 M65ED levels is an independent risk factor for cardiometabolic disorders, after accounting for general cardiometabolic risk parameters or the presence of NAFLD. Additionally, we found that the prevalence of cardiometabolic disorders and its components increased with increasing quartiles of CK18 M65ED.

Several studies have been conducted to assess serum CK18 levels in NAFLD, as it is one of the most extensively evaluated biomarkers of steatohepatitis [35]. However, to our knowledge, limited studies have examined the association between serum CK18 M65ED levels and the risk for cardiometabolic disorders. In accordance with the results of Tan et al. and Civera et al., we showed that CK18 M65ED is positively correlated with the insulin resistance index of HOMA-IR [20, 21]; however, the correlation was not observed in the multiple stepwise regression analysis. The CK18 M65ED levels positively correlated with fasting glucose levels, and it was associated with cardiometabolic disorders independent of NAFLD status, which is in agreement with the results of Chang et al. [19]. In addition to adult hepatocytes [16], CK18 can be expressed in simple epithelial cells, cholangiocytes, pancreas, and colon [16, 24, 25]. CK18 M65ED can act as a cell death marker in cardiometabolic disorders because of its ability to detect both native and intact CK18 [18]. Previous studies have demonstrated that apoptosis can cause a decrease in the number of circulating mucosal-associated invariant T cells in cardiometabolic diseases [7]. Moreover, myocardial cells undergo death in obesity-associated cardiomyopathy in high-fat diet-fed mice [8]. In the present study, we extend these investigations by providing further evidences of the possibility of cell death in cardiometabolic disorders.

The strong correlation between serum CK18 M65ED levels and the risk for cardiometabolic disorders might be explained by the interactions among various metabolic stress inducers that are activated by nutritional surplus, inflammation, innate immune system, and gut microbiota during the induction of cell death. Under conditions of glucotoxicity, lipotoxicity, and exposure to proinflammatory cytokines or biologically active sphingolipids, cell death mediated by ER stress, 4-hydroxynonenal, and endogenous reactive oxygen species may contribute to the pathogenesis of cardiometabolic disorders [9, 10, 36]. Long-lasting and low-grade chronic inflammation mediated by cytokines and chemokines, which are primarily released by innate immune cells under cardiometabolic stress, is a distinguishing feature of cardiometabolic disorders, and their overactive responses can directly contribute to the pathogenesis of such diseases [37–39]. For example, pancreatic *β*-cell apoptosis due to hyperglycemia and ER stress can contribute to the decreased *β*-cell mass that characterizes T2D, and the activation of inflammasomes by these harmful factors can lead to *β*-cell death [9]. Thus, *β*-cell death and cell death-associated inflammation interact with each other through innate immune receptors [9]. Another explanation might be that altered microbial compositions in patients with cardiometabolic disorders may foster inflammation by producing more proinflammatory molecules such as lipopolysaccharide and peptidoglycans that can interact with host pattern recognition receptors of the innate immune system [40–42]. Winer et al. have demonstrated that obesity-altered gut microbiome can increase the recruitment of macrophages and reduce the proportion of regulatory T cells within the gut [43]. Touch et al. have suggested that apoptosis of mucosal-associated invariant T cells in patients with cardiometabolic disorders occurs due to gut microbiome dysbiosis and enhanced proapoptotic signaling, which is caused by long-term and low-grade systemic inflammation [7]. The pathway in which CK18 is involved in cell death at a molecular level is, however, not very clear. CK18 could regulate the transcription of apoptotic genes *FAS* and *FADD*, as well as immune genes *CXCL2* and *CD79B*, in addition to alternative splicing of *FAS* and *CTNNB1* [13]. More precisely designed studies should be conducted to isolate peripheral blood mononuclear cells from the whole blood of the subjects and measure the expression of cell death-related genes and proteins to explore why CK18 M65ED is positively associated with cardiometabolic disorders.

Recent studies demonstrated that some serum metabolites, ratios of several traditional metabolic parameters, and adipokines could have closed relationship with the cardiometabolic risk. Serum glycans could improve type 2 diabetes and CVD prediction beyond established risk markers [44]. TG : HDL-C ratio and triglyceride-glucose index are all reported to be associated with the cardiometabolic disorders [45, 46]. Leptin, adiponectin, and their ratio were associated with insulin resistance and other cardiometabolic comorbidities [47]. In the latest researches, branched chain amino acids were found to be passive biomarkers or the key to the pathogenesis of cardiometabolic diseases [48]. Based on our data, instead of ratios and in an easier testing method, CK18 M65ED is an independent risk factor for cardiometabolic disorders, which remained statistically significant after adjustment with age, gender, NAFLD, and traditional metabolic variables.

The limitation of this study is its cross-sectional study design and relatively small sample size, which made it difficult to address the cause-effect relationship between serum CK18 M65ED levels and cardiometabolic disorders. Secondly, the serum levels of CK18 M65ED provided limited information about the cell death condition in the body. Hence, further prospective studies are warranted to confirm the present findings in a larger population.

## 5. Conclusions

In this study, the serum levels of CK18 M65ED, as a cell death marker, were significantly increased among subjects with cardiometabolic disorders, and these increased levels were associated with the risk for cardiometabolic disorders, independent of other cardiometabolic risk parameters, and NAFLD. Our study suggested that elevated serum CK18 M65ED levels could be used to predict the risk for cardiometabolic disorders.

## Figures and Tables

**Table 1:** Characteristics of subjects with and without cardiometabolic disorders.

Variables	Total	Without cardiometabolic disorders	With cardiometabolic disorders	*P* value without CD vs. with CD
*n* (male/female)	588 (340/248)	278 (109/169)	310 (231/79)	<0.001
Age (years)	40.0 (33.0–49.8)	40.0 (32.0–47.0)	40.0 (34.0–52.0)	0.101
Body mass index (kg/m^2^)^§^	24.8 (22.1–27.9)	22.2 (20.4–23.6)	27.7 (25.4–30.4)	<0.001
Waist circumference (cm)^§^	86.5 (72.0–97.5)	71.0 (66.0–77.0)	96.7 (90.6–103.0)	<0.001
Systolic blood pressure (mmHg)^§^	118 (110–124)	114 (109–120)	120 (112–128)	<0.001
Diastolic blood pressure (mmHg)^§^	80 (72–82)	76 (70–80)	80 (74–85)	<0.001
Fasting plasma glucose (mmol/L)^§^	5.08 (4.72–5.58)	4.96 (4.63–5.27)	5.28 (4.84–5.98)	0.251
2-hour plasma glucose (mmol/L)^§^	6.69 (5.61–8.12)	5.69 (4.93–6.58)	7.71 (6.70–9.82)	<0.001
HbA1c (%)^§^	5.6 (5.4–5.9)	5.5 (5.3–5.7)	5.7 (5.4–6.3)	0.005
Fasting insulin (*μ*U/mL)^§^	10.10 (5.28–13.10)	5.51 (3.93–8.51)	12.65 (10.99–16.73)	<0.001
HOMA-IR^§^	2.26 (1.18–3.23)	1.21 (0.85–1.89)	3.10 (2.49–4.39)	<0.001
HOMA-B^§^	30.57 (16.92–49.4)	21.81 (13.88–34.93)	41.04 (28.37–64.9)	0.001
Alanine aminotransferase (IU/L)^§^	21 (13–33)	14 (10.75–19.00)	29 (20–43)	<0.001
Aspartate transaminase (IU/L)^§^	20 (17–25)	18 (16–21)	22 (19–28)	<0.001
Gamma-glutamyl-transferase (IU/L)^§^	24 (15–40)	16 (13–24)	37 (24–49)	<0.001
Triglycerides (mmol/L)^§^	1.31 (0.89–1.86)	1.01 (0.72–1.30)	1.74 (1.28–2.38)	<0.001
Total cholesterol (mmol/L)	4.75 ± 0.86	4.42 ± 0.73	5.04 ± 0.85	<0.001
HDL cholesterol (mmol/L)^§^	1.21 (1.05–1.4)	1.37 (1.23–1.57)	1.08 (0.95–1.21)	<0.001
LDL cholesterol (mmol/L)	2.94 ± 0.73	2.73 ± 0.62	3.12 ± 0.77	<0.001
Cytokeratin 18 M65ED (U/L)^§^	137.54 (76.51–254.39)	83.85 (52.80–153.75)	197.36 (121.13–354.50)	<0.001

Data are mean ± SD or median (interquartile range). ^§^Log transformed before analysis. CD: cardiometabolic disorders; HbA1c: glycated hemoglobin A1c; HDL: high-density lipoprotein; LDL: low-density lipoprotein.

**Table 2:** Characteristics of subjects and prevalence of cardiometabolic disorders and individual components according to CK18 M65ED quartiles.

Variables	Serum CK18 M65ED quartiles				*P* for trend
	Quartile 1	Quartile 2	Quartile 3	Quartile 4	
*n* (male/female)	71/76	74/73	90/57	105/42	—
Age (years)^§^	39 (33–45)	42 (34–52)	40 (34–48)	38 (32–52)	0.159
Body mass index (kg/m^2^)^§^	22.8 (20.8–24.5)	24.1 (21.8–26.6)	25.9 (22.9–29.3)	27.4 (24.5–30.5)	<0.001
Waist circumference (cm)^§^	76.0 (67.0–86.0)	81.0 (71.0–95.0)	90.1 (73.0–100.0)	95.0 (87.6–102.9)	<0.001
Systolic blood pressure (mmHg)^§^	112.0 (108.5–120.0)	116.0 (109.0–124.0)	119.0 (110.0–125.0)	120.0 (114.0–128.5)	<0.001
Diastolic blood pressure (mmHg)^§^	75 (70–80)	78 (71–80.5)	80 (73.8–84)	80 (76–85)	<0.001
Fasting plasma glucose (mmol/L)^§^	4.94 (4.61–5.3)	5.06 (4.72–5.49)	5.07 (4.79–5.68)	5.33 (4.90–6.14)	<0.001
2-hour plasma glucose (mmol/L)^§^	5.98 (5.16–7.21)	6.56 (5.44–8.05)	6.800 (5.67–8.18)	7.72 (6.49–10.42)	<0.001
HbA1c (%)^§^	5.5 (5.3–5.7)	5.55 (5.3–5.8)	5.6 (5.4–5.8)	5.7 (5.4–6.8)	<0.001
Fasting insulin (*μ*U/mL)^§^	6.50 (4.60–11.58)	8.40 (5.09–12.30)	10.69 (5.24–13.79)	11.90 (10.16–16.71)	<0.001
HOMA-IR^§^	1.42 (1.02–2.48)	1.80 (1.11–2.86)	2.44 (1.16–3.44)	2.94 (2.38–4.78)	<0.001
HOMA-B^§^	26.21 (16.27–48.58)	29.41 (15.47–43.94)	30.97 (15.91–57.90)	36.34 (22.78–55.74)	0.078
Alanine aminotransferase (IU/L)^§^	14 (11–20)	16 (11.75–23)	22 (14–35)	41 (24–62)	<0.001
Aspartate transaminase (IU/L)^§^	18 (16–21)	18 (16–22)	21 (17–25)	27 (21–32)	<0.001
Gamma-glutamyl-transferase (IU/L)^§^	17 (13–26)	19.5 (14–28)	31 (17–41)	40 (24–56)	<0.001
Triglycerides (mmol/L)^§^	1.08 (0.77–1.45)	1.17 (0.79–1.69)	1.49 (1.03–2.15)	1.74 (1.14–2.38)	<0.001
Total cholesterol (mmol/L)	4.48 ± 0.81	4.69 ± 0.84	4.82 ± 0.84	4.99 ± 0.85	<0.001
HDL cholesterol (mmol/L)^§^	1.31 (1.20–1.48)	1.28 (1.11–1.47)	1.13 (0.97–1.35)	1.10 (0.95–1.21)	<0.001
LDL cholesterol (mmol/L)	2.77 ± 0.70	2.90 ± 0.69	2.97 ± 0.72	3.12 ± 0.76	<0.001
Cytokeratin 18 M65 (U/L)^§^	53.00 (43.08–62.25)	104.69 (87.88–120.78)	175.22 (155.98–210.06)	412.52 (321.94–594.20)	<0.001
Cardiometabolic disorders (*n*, %)	29 (19.73%)	70 (47.62%)	90 (61.22%)	121 (82.31%)	<0.001
Obesity (*n*, %)	4 (2.72%)	11 (7.48%)	28 (19.05%)	45 (30.61%)	<0.001
Central obesity (*n*, %)	25 (17.01%)	59 (40.14%)	81 (55.1%)	107 (72.79%)	<0.001
Diabetes (*n*, %)	1 (0.68%)	14 (9.52%)	19 (12.93%)	42 (28.57%)	<0.001
Dyslipidemia (*n*, %)	2 (1.36%)	29 (19.73%)	65 (44.22%)	78 (53.06%)	<0.001
Hypertension (*n*, %)	1 (0.68%)	12 (8.16%)	20 (13.61%)	38 (25.85%)	<0.001
No. of CD components					
One or more (*n*, %)	29 (19.73%)	70 (47.62%)	90 (61.22%)	122 (82.99%)	<0.001
Two or more (*n*, %)	0 (0%)	29 (19.73%)	67 (45.58%)	90 (61.22%)	<0.001
Three or more (*n*, %)	0 (0%)	11 (7.48%)	22 (14.97%)	38 (25.85%)	<0.001
Four (*n*, %)	0 (0%)	4 (2.72%)	6 (4.08%)	15 (10.2%)	<0.001

^§^Log transformed before analysis. CK18: cytokeratin 18; CD: cardiometabolic disorder.

**Table 3:** Pearson correlation analysis and multivariate stepwise regression analysis for serum CK18 M65ED levels.

Variables	Pearson correlation analysis		Multiple stepwise regression analysis		
	*r*	*P* value	Standardized *β*	*t*	*P* value^a^
Gender	-0.16	<0.001	—	—	—
Age (years)	0.01	0.806	—	—	—
Body mass index (kg/m^2^)^§^	0.404	<0.001	—	—	—
Waist circumference (cm)^§^	0.401	<0.001	—	—	—
Systolic blood pressure (mmHg)^§^	0.303	<0.001	—	—	—
Diastolic blood pressure (mmHg)^§^	0.315	<0.001	0.207	5.279	<0.001
Fasting plasma glucose (mmol/L)^§^	0.26	<0.001	—	—	—
2-hour plasma glucose (mmol/L)^§^	0.323	<0.001	—	—	—
HbA1c (%)^§^	0.234	<0.001	0.171	4.526	<0.001
Fasting insulin (*μ*U/mL)^§^	0.249	<0.001	—	—	—
HOMA-IR^§^	0.297	<0.001	—	—	—
HOMA-B^§^	0.081	0.06	—	—	—
Alanine aminotransferase (IU/L)^§^	0.522	<0.001	0.474	10.464	<0.001
Aspartate transaminase (IU/L)^§^	0.431	<0.001	—	—	—
Gamma-glutamyl-transferase (IU/L)^§^	0.388	<0.001	—	—	—
Triglycerides (mmol/L)^§^	0.329	<0.001	—	—	—
Total cholesterol (mmol/L)	0.199	<0.001	—	—	—
HDL cholesterol (mmol/L)^§^	-0.339	<0.001	-0.135	-3.035	0.003
LDL cholesterol (mmol/L)	0.153	<0.001	—	—	—

^§^Log transformed before analysis. ^a^Original variables included all the variables with *P* < 0.05 in the Pearson correlation analysis and were adjusted for gender. CK18: cytokeratin 18; HbA1c: glycated hemoglobin A1c; HDL: high-density lipoprotein; LDL: low-density lipoprotein.

**Table 4:** Odds ratios of cardiometabolic disorders based on serum cytokeratin 18 M65ED as a continuous or categorical variable.

	Odds ratios (95% CI)							
	Model 1^a^	*P*	Model 2^b^	*P*	Model 3^c^	*P*	Model 4^d^	*P*
M65ED as categories								
Quartile 1	1.00 (1.00–1.00)	—	1.00 (1.00–1.00)	—	1.00 (1.00–1.00)	—	1.00 (1.00–1.00)	—
Quartile 2	2.77 (1.31–5.85)	0.008	3.41 (1.13–10.29)	0.03	3.24 (1.00–10.44)	0.049	4.02 (2.31–7.03)	<0.001
Quartile 3	3.14 (1.43–6.89)	0.004	3.74 (1.00–13.95)	0.049	4.96 (1.20–20.47)	0.027	6.64 (3.79–11.65)	<0.001
Quartile 4	9.08 (3.91–21.08)	<0.001	14.28 (2.79–73.21)	0.001	16.83 (2.75–103.19)	0.002	18.39 (9.89–34.21)	<0.001
*P* for trend		<0.001		0.012		0.018		<0.001
M65ED (per 1 SD)	1.92 (1.39–2.65)	<0.001	1.90 (1.13–3.19)	0.015	1.97 (1.11–3.48)	0.020	2.75 (2.07–3.66)	<0.001

^a^Model 1 adjusted for age, gender, and BMI. ^b^Model 2 adjusted for variables in model 1 and also for SBP, DBP, ALT, AST, GGT, TG, TC, HDL-C, LDL-C, HOMA-IR, HOMA-B, and HbA1c. ^c^Model 3 adjusted for variables in model 1 and also for SBP, DBP, ALT, AST, GGT, TG, TC, HDL-C, LDL-C, FPG, 2hPG, HbA1c, and FINS. ^d^Model 4 adjusted for age, gender, and nonalcoholic fatty liver disease (yes, no). Serum cytokeratin 18 M65ED levels: quartile 1: ≤76.51 U/L; quartile 2: 76.51–137.54 U/L; quartile 3: 137.54–254.39 U/L; quartile 4: ≥254.39 U/L. CI: confidence interval; SD: standard deviation.

## Data Availability

The data used to support the findings of this study are available from the corresponding author upon request.
